# Role of tumor necrosis factor–α and its receptors in diesel exhaust particle-induced pulmonary inflammation

**DOI:** 10.1038/s41598-017-11991-7

**Published:** 2017-09-14

**Authors:** Smitha Kumar, Guy Joos, Louis Boon, Kurt Tournoy, Sharen Provoost, Tania Maes

**Affiliations:** 1 Laboratory for Translational Research in Obstructive Pulmonary Diseases - Department of Respiratory Medicine, Ghent University Hospital, Ghent, Belgium; 2 Bioceros B.V., Utrecht, Netherlands

## Abstract

Inhalation of diesel exhaust particles (DEP) induces an inflammatory reaction in the lung. However, the underlying mechanisms remain to be elucidated. Tumor necrosis factor alpha (TNF-α) is a pro-inflammatory cytokine that operates by binding to tumor necrosis factor receptor 1 (TNFR1) and tumor necrosis factor receptor 2 (TNFR2). The role of TNF-α signaling and the importance of either TNFR1 or TNFR2 in the DEP-induced inflammatory response has not yet been elucidated. TNF-α knockout (KO), TNFR1 KO, TNFR2 KO, TNFR1/TNFR2 double KO (TNFR-DKO) and wild type (WT) mice were intratracheally exposed to saline or DEP. Pro-inflammatory cells and cytokines were assessed in the bronchoalveolar lavage fluid (BALF). Exposure to DEP induced a dose-dependent inflammation in the BALF in WT mice. In addition, levels of TNF-α and its soluble receptors were increased upon exposure to DEP. The DEP-induced inflammation in the BALF was decreased in TNF-α KO, TNFR-DKO and TNFR2 KO mice. In contrast, the inflammatory response in the BALF of DEP-exposed TNFR1 KO mice was largely comparable with WT controls. In conclusion, these data provide evidence for a regulatory role of TNF-α in DEP-induced pulmonary inflammation and identify TNFR2 as the most important receptor in mediating these inflammatory effects.

## Introduction

In the last decades, several epidemiological studies have shown an association between inhalation of particulate matter pollutants and adverse pulmonary health effects including increased risk for respiratory tract infections, asthma, chronic bronchitis, chronic obstructive pulmonary disease and lung cancer. Particulate matter emissions from diesel engines (diesel exhaust particles, DEP) are a major contributor to the ambient air pollution problem^1, 2^. In controlled human exposure studies, inhalation of diesel exhaust or DEP was associated with increased airway resistance and airway inflammation in healthy individuals; as demonstrated by increased neutrophils, lymphocytes and mast cells and elevated IL-8, IL-6 and myeloperoxidase levels in bronchoalveolar lavage fluid (BALF), bronchial biopsies and sputum^3–6^. Also in murine studies, we have previously demonstrated that exposure to DEP induces an inflammatory response in the lung characterized by the accumulation of neutrophils, monocytes, dendritic cells and T-cells^7, 8^. Moreover, DEP exposure increased pro-inflammatory cytokine and chemokine expression in BALF and lung tissue^9^. However, the precise molecular and cellular events underlying the DEP-induced pulmonary inflammation remain unclear. Understanding these mechanisms could provide new tools to protect and treat at-risk individuals from the adverse effects of particulate air pollutants.

Tumor necrosis factor alpha (TNF-α) is a pleiotropic cytokine and is an important mediator of inflammation. It is generated as a type II transmembrane protein that can be proteolytically processed by TNF-converting enzyme (TACE or ADAM17) into a soluble form. Both the transmembrane and soluble protein mediate their biological effects by binding to TNF receptor 1 (TNFR1, p55) and TNF receptor 2 (TNFR2, p75), which differ in structure, expression patterns and signaling pathways that they induce^10^.

The contribution of TNF-α/TNFR signaling to the DEP-induced lung inflammation remains poorly understood and controversial. Whereas several studies reported that in vitro stimulation with particulate matter and DEP resulted in increased TNF-α secretion from lung epithelial cells, alveolar macrophages and peripheral blood mononuclear cells from human or mice^11–13^, others described no or rather a decreased TNF-α release from macrophages in response to DEP^14^. Interestingly, increased TNF-α levels in plasma were found in healthy individuals that were exposed to diesel exhaust^15^. In animal studies, acute and chronic exposure to DEP was reported to be associated with elevated TNF-α levels in lung tissue^16–19^, however single or multiple DEP exposures up to 4 days in TNF-α knockout (KO) mice showed no role for TNF-α in the acute DEP-induced neutrophilic response^17, 20^. The involvement of TNF-signaling in other DEP-induced inflammatory cell responses has not yet been addressed thus far.

In this manuscript, we examined the role of TNF-α/TNFR signaling in DEP-induced lung inflammation. For that, we evaluated TNF-α knockout (KO) mice as well as TNFR1 KO, TNFR2 KO and TNFR1/TNFR2 double KO (TNFR-DKO) mice in an established murine model of DEP-induced pulmonary inflammation.

## Results

### Exposure to DEP induces inflammation in BALF in a dose-dependent manner

**Figure 1:**
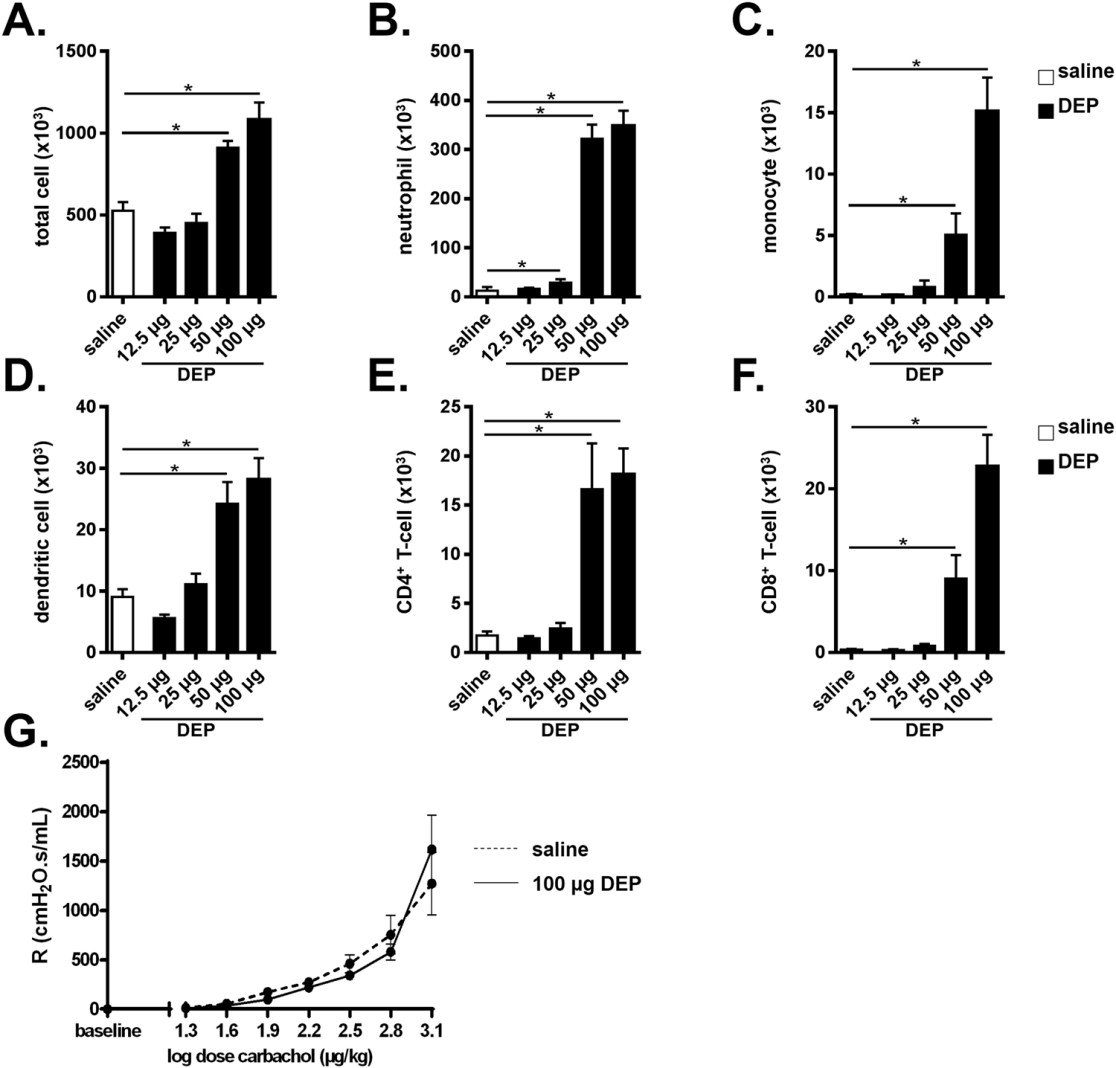
Exposure to DEP induces inflammation in BALF in a dose-dependent manner. C57BL/6 J WT mice were exposed to saline (white bar) or 12.5 µg, 25 µg, 50 µg and 100 µg DEP (black bars). BALF was collected 48 hours after the last exposure. (**A**) Total cell numbers in BALF. (**B**) Neutrophils in BALF were determined on cytospin. (**C–F**) Monocyte (CD11c^low^, CD11b^+^, Ly6C^+^ and Ly6G^−^) **(C)**; dendritic cell (CD11c^high^, low autofluorescent and MHCII^+^) **(D)**; CD4^+^ T-cell (CD3^+^, CD8^−^, CD4^+^) **(E)** and CD8^+^ T-cell (CD3^+^, CD4^−^, CD8^+^) **(F)** numbers in BALF were determined by flow cytometry. (**G**) Airway resistance (R) of saline- (broken line) and 100 µg DEP- (full line) exposed mice was measured in response to increasing doses of carbachol. Results are expressed as mean ± SEM. n = 8 mice per group. *p < 0.05.

C57BL/6 J wild type (WT) mice were exposed to saline and increasing doses of DEP (i.e. 12.5 µg, 25 µg, 50 µg or 100 µg) and analyzed at day 9. Exposure to DEP increased the number of total BALF cells, including neutrophils, monocytes, dendritic cells, CD4^+^ T-cells and CD8^+^ T-cells in a dose-dependent manner (Fig. 1A–F). When compared with the saline-exposed group, statistically significant increased BALF inflammatory cell numbers were mainly observed in the groups exposed to 50 µg DEP and 100 µg DEP (Fig. 1A–F). Since the DEP-induced inflammation was more pronounced with the 100 µg DEP dose, we selected this dose for the further experiments described below.

Exposure to DEP has been associated with increased airway hyperresponsiveness (AHR)^21^. To examine this in our murine model, lung function was measured in WT mice that were exposed to saline and 100 µg DEP by assessing AHR to carbachol. As shown in Fig. 1G, there was no difference in airway responsiveness between saline and DEP-exposed mice.

### TNF-α and its soluble receptors are increased in WT mice exposed to DEP

**Figure 2:**
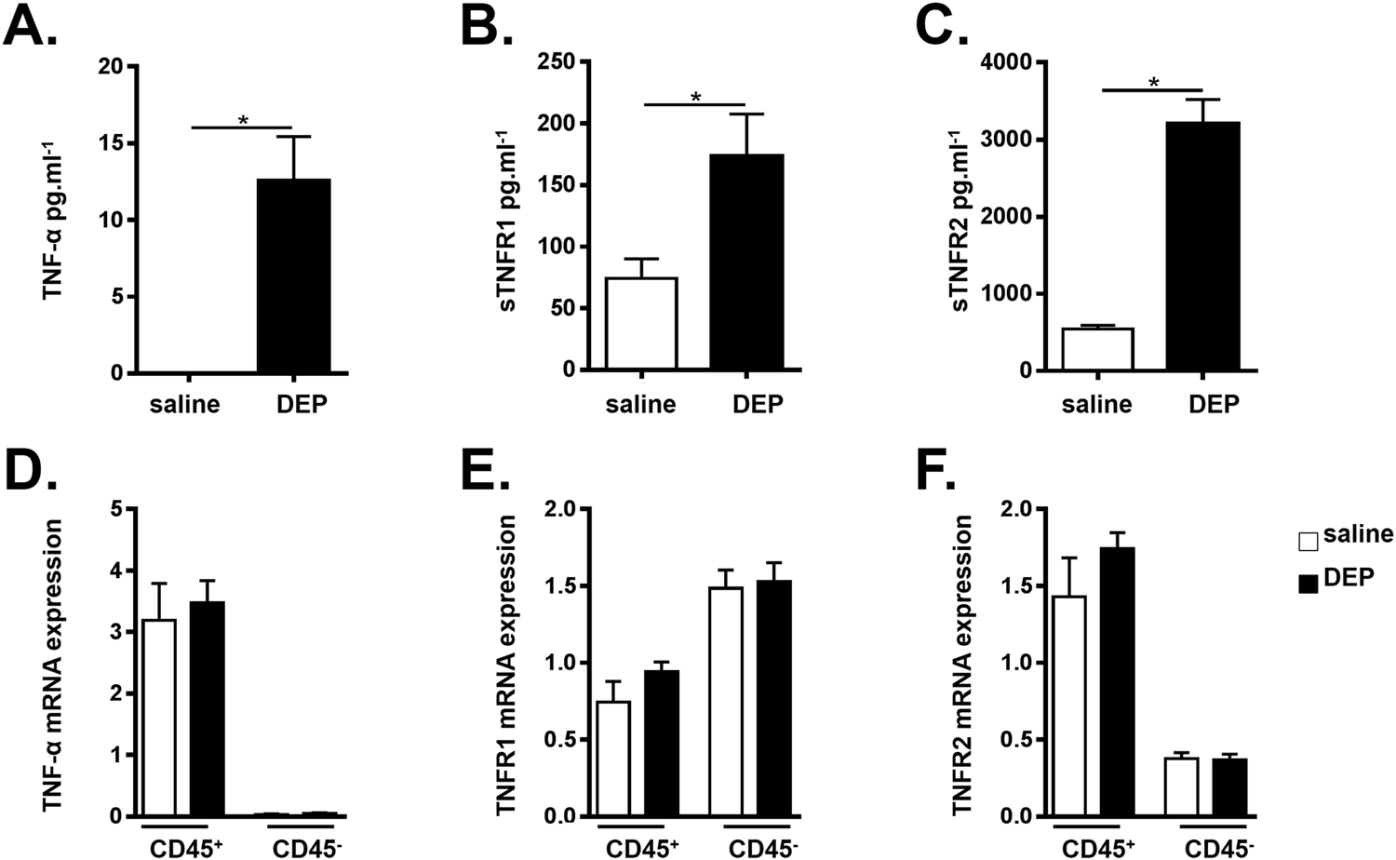
TNF-α and its soluble receptors are increased in WT mice exposed to DEP. C57BL/6 J WT mice were exposed to saline (white bar) or 100 µg DEP (black bar). BALF and lung tissue were collected 48 hours after the last exposure. (**A–C**) TNF-α **(A)**, sTNFR1 **(B)** and sTNFR2 **(C)** levels were determined in BALF using ELISA. Results are expressed as mean ± SEM. n = 7–9 mice per group. *p < 0.05. Data are representative of two independent experiments. (**D–F**) TNF-α **(D)**, TNFR1 **(E)** and TNFR2 **(F)** mRNA expression in hematopoietic (CD45^+^) and non-hematopoietic (CD45^−^) lung cells were determined using qRT-PCR. n = 5–8 mice per group.

Using ELISA, we assessed whether *in vivo* exposure to DEP induces a TNF-α response in the lungs. Exposure to 100 µg DEP was associated with significantly elevated TNF-α levels in BALF in comparison with saline-exposed mice (Fig. 2A). The same analysis at 3, 6, 12 or 24 hours revealed no change in TNF-α levels (data not shown).

The biological responses of TNF-α are mediated by its two receptors TNFR1 and TNFR2, both of which can be released as soluble molecules. As shown in Fig. 2B,C, exposure to DEP elevated soluble (s)TNFR1 and sTNFR2 levels in BALF when compared with the saline-treated group.

To examine the distribution and expression of TNF-α, TNFR1 and TNFR2 in our model, we sorted hematopoietic cells (CD45^+^) versus non-hematopoietic (CD45^−^) cells from lung tissue of saline and DEP-exposed WT mice, and examined the mRNA expression. We found that TNF-α mRNA was preferentially expressed in the CD45^+^ hematopoietic lung cell population, while limited TNF-α mRNA expression was seen in the CD45^−^ lung cell population (Fig. 2D). TNFR2 mRNA was also predominantly expressed in the CD45^+^ lung cell population when compared with the CD45^−^ cell fraction (Fig. 2F), whereas TNFR1 mRNA expression was the highest in CD45^−^ cells (Fig. 2E). In these sorted cell populations, we found no difference in TNF-α, TNFR1 and TNFR2 mRNA expression between saline and DEP-exposed mice.

### DEP-induced pulmonary inflammation is TNF-α dependent

**Figure 3:**
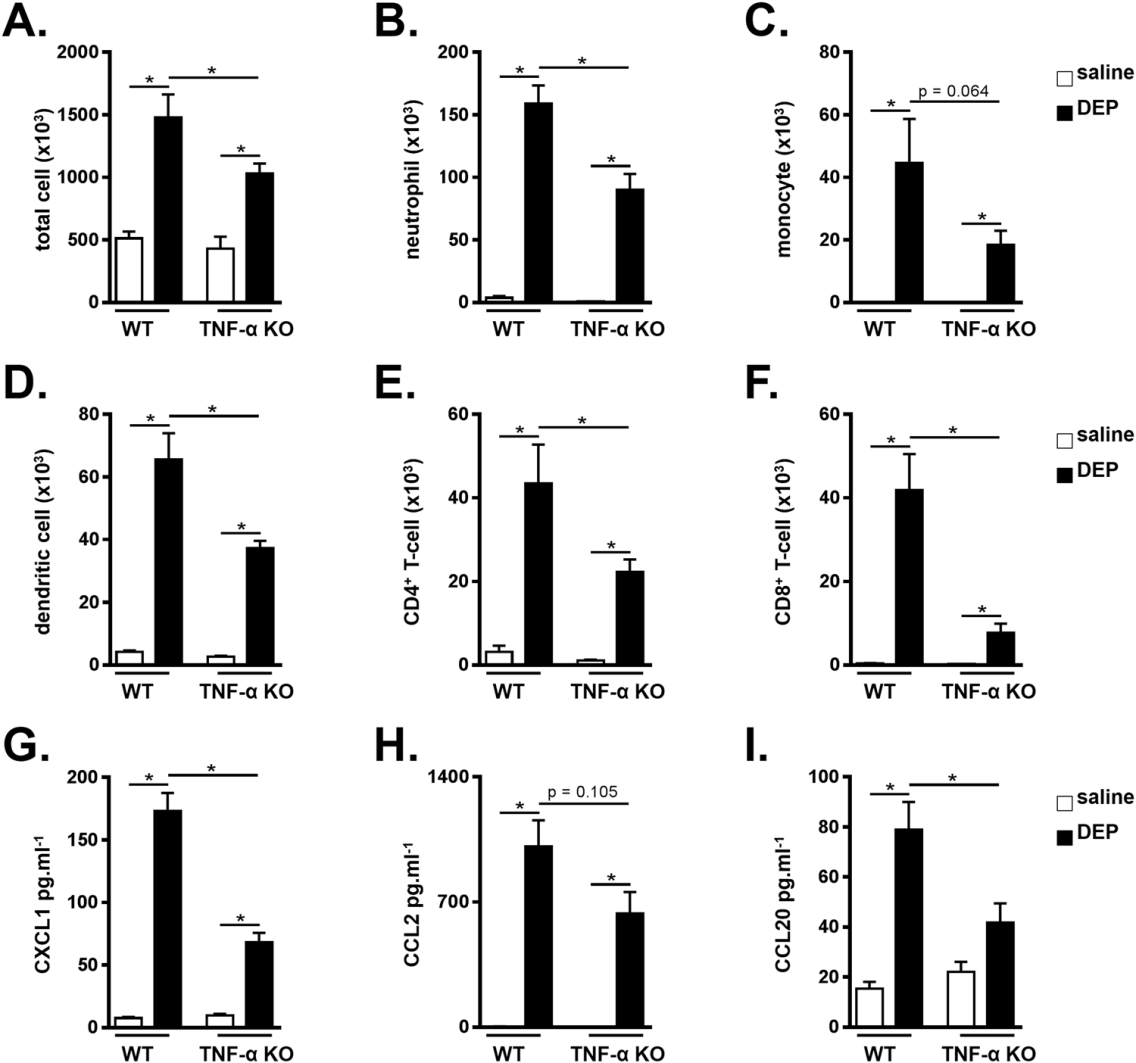
DEP-induced inflammation is TNF-α dependent. C57BL/6 J WT and TNF-α KO were exposed to saline (white bar) or 100 µg DEP (black bar). BALF was collected 48 hours after the last exposure. (**A**) Total cell numbers in BALF. (**B–F**) Neutrophil (CD11c^low^, CD11b^+^, Ly6C^+^ and Ly6G^+^) **(B)**; monocyte (CD11c^low^, CD11b^+^, Ly6C^+^ and Ly6G^−^) **(C)**; dendritic cell (CD11c^high^, low autofluorescent and MHCII^+^) **(D)**; CD4^+^ T-cell (CD3^+^, CD8^−^, CD4^+^) **(E)** and CD8^+^ T-cell (CD3^+^, CD4^−^, CD8^+^) **(F)** numbers in BALF were determined by flow cytometry. (**G–I**) CXCL1 **(G)**, CCL2 **(H)** and CCL20 **(I)** levels were determined in BALF using ELISA. Results are expressed as mean ± SEM. n = 5–8 mice per group. *p < 0.05.

To assess the functional contribution of TNF-α to DEP-induced pulmonary inflammation, WT and TNF-α KO mice were exposed to saline or 100 µg DEP and analyzed at day 9. Upon exposure to DEP, TNF-α KO mice had reduced numbers of total BALF cells, including neutrophils, dendritic cells, CD4^+^ T-cells and CD8^+^ T-cells when compared with the DEP-exposed WT mice (Fig. 3A,B,D–F respectively). BALF monocytes only tended to decrease in DEP-exposed TNF-α KO mice in comparison with WT mice that received DEP (Fig. 3C).

Exposure to DEP in WT mice elevated the levels of pro-inflammatory cytokines chemokine CXC ligand-1 (CXCL1), CC chemokine ligand 2 (CCL2), and CC chemokine ligand 20 (CCL20) in BALF (Fig. 3G–I). DEP-exposed TNF-α KO mice however showed an impaired increase in CXCL1 and CCL20 (Fig. 3G and I respectively). The DEP-induced CCL2 levels tended to decrease in TNF-α KO mice compared to WT mice, although this was not statistical significant.

### DEP-induced pulmonary inflammation is dependent on TNF receptor signaling

**Figure 4:**
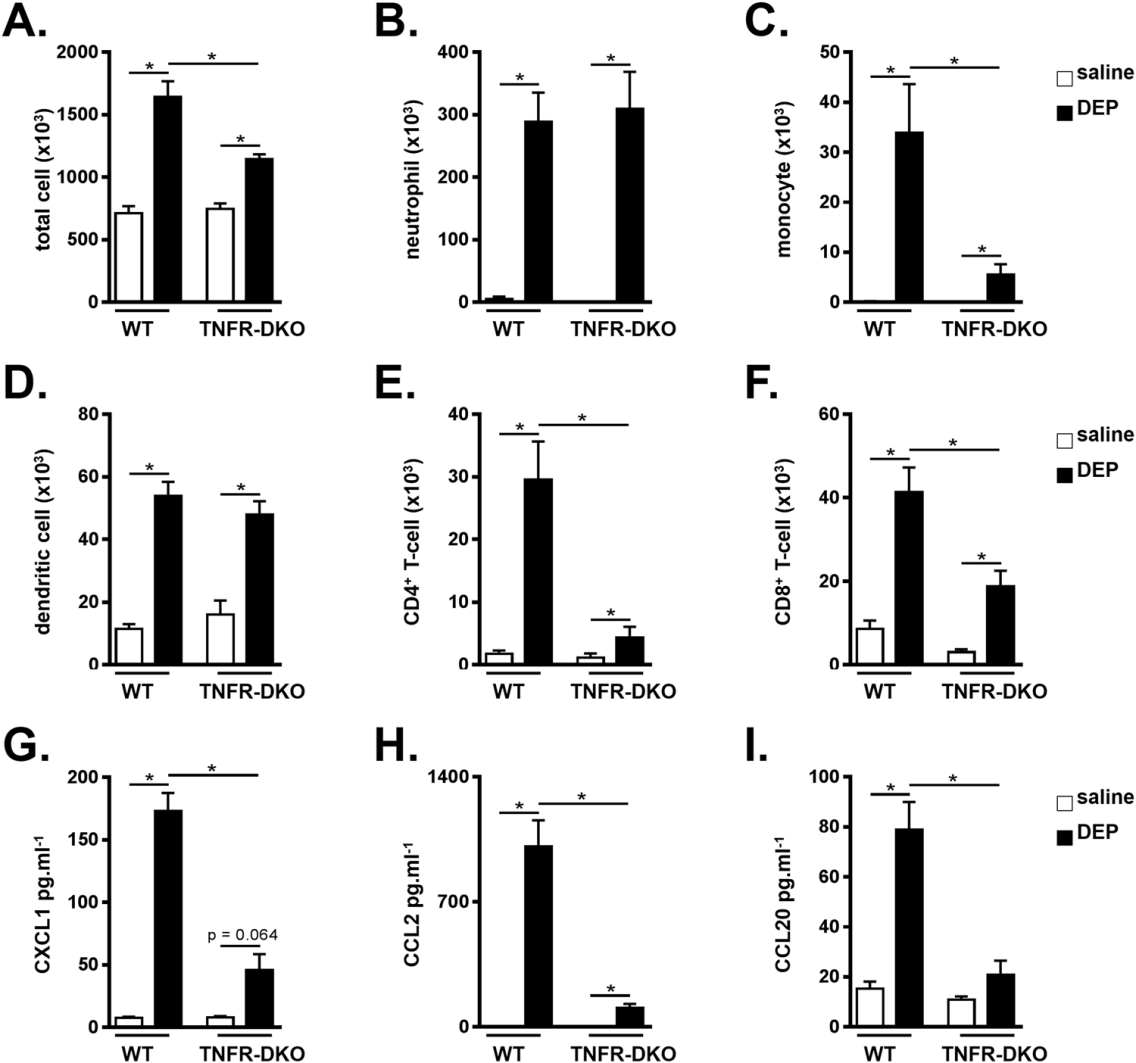
DEP-induced pulmonary inflammation is dependent on TNF receptor signaling. C57BL/6 J WT and TNFR-DKO mice were exposed to saline (white bar) or 100 µg DEP (black bar). BALF was collected 48 hours after the last exposure. (**A**) Total cell numbers in BALF. (**B–F**) Neutrophil (CD11c^low^, CD11b^+^, Ly6C^+^ and Ly6G^+^) **(B)**; Monocyte (CD11c^low^, CD11b^+^, Ly6C^+^ and Ly6G^−^) **(C)**; dendritic cell (CD11c^high^, low autofluorescent and MHCII^+^) **(D)**; CD4^+^ T-cell (CD3^+^, CD8^−^, CD4^+^) **(E)** and CD8^+^ T-cell (CD3^+^, CD4^−^, CD8^+^) **(F)** numbers in BALF were determined by flow cytometry. (**G–I**) CXCL1 **(G)**, CCL2 **(H)** and CCL20 **(I)** levels were determined in BALF using ELISA. Results are expressed as mean ± SEM. n = 7–8 mice per group. *p < 0.05. BALF inflammatory cell data are representative of two independent experiments (except for BALF neutrophils).

We next examined the contribution of TNF receptor signaling in the model of DEP-induced pulmonary inflammation. For that, TNFR-DKO, which lack functional TNFR1 and TNFR2, and WT control mice were exposed to saline or 100 µg DEP. TNFR-DKO showed a decrease in DEP-induced total BALF cells, BALF monocytes, CD4^+^ T-cells and CD8^+^ T-cells in comparison with WT controls (Fig. 4A,C,E and F). In contrast, the increase in neutrophils and dendritic cells in the BALF was similar between DEP-treated TNFR-DKO mice and WT controls (Fig. 4B and D).

Deficiency in TNF receptor signaling also resulted in impaired levels of CXCL1, CCL2 and CCL20 in the BALF of DEP-exposed TNFR-DKO mice, when compared with DEP-treated WT controls (Fig. 4G–I). In contrast to WT animals, the levels of CXCL1 and CCL20 did not significantly increase in DEP-exposed TNFR-DKO mice when compared to the saline controls.

### DEP-induced monocytes and lymphocytes are TNFR2 dependent

**Figure 5:**
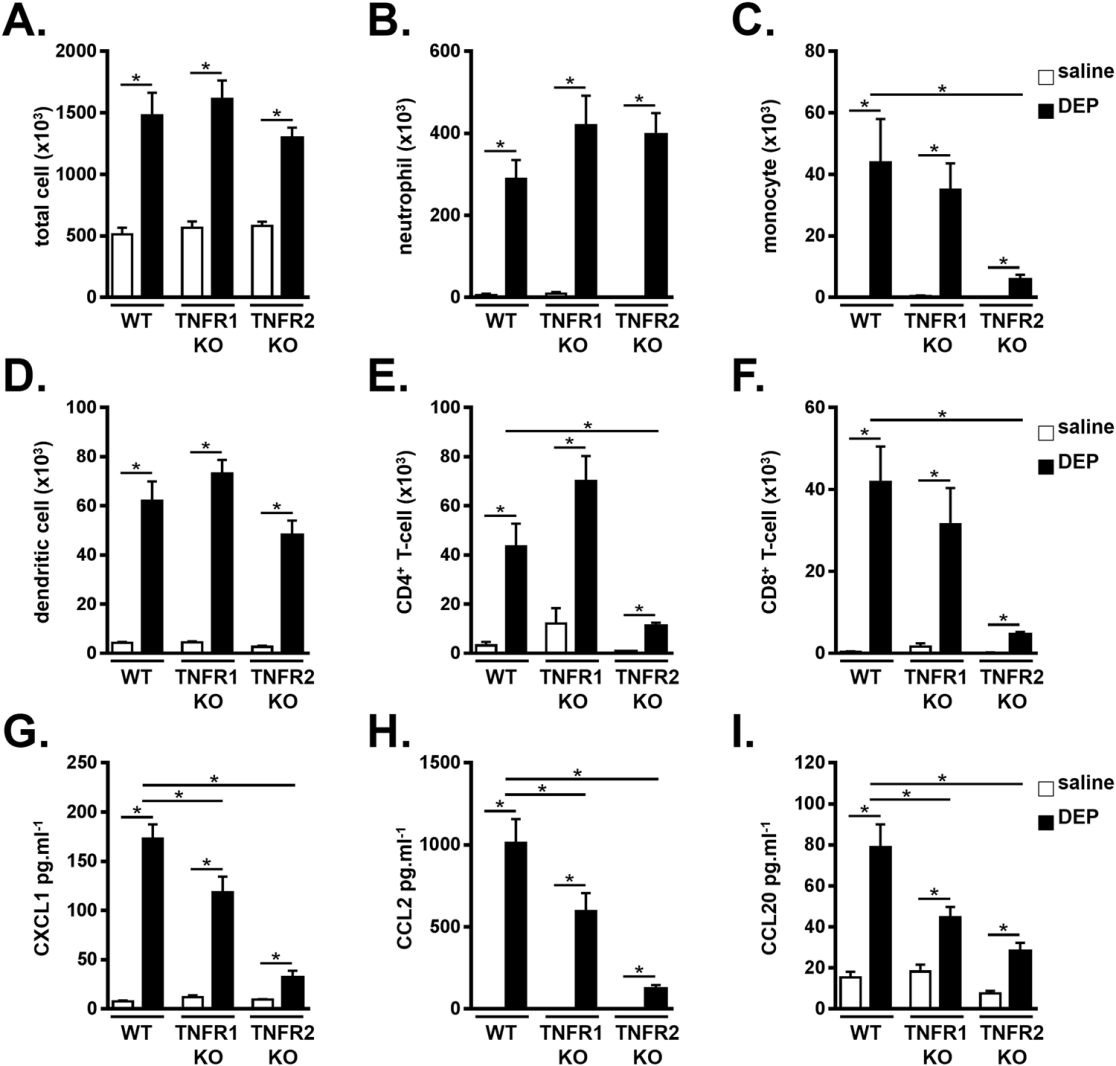
DEP-induced monocytes and lymphocytes are TNFR2 dependent. C57BL/6 J WT, TNFR1 KO and TNFR2 KO mice were exposed to saline (white bar) or 100 µg DEP (black bar). BALF was collected 48 hours after the last exposure. (**A**) Total cell numbers in BALF. (**B–F**) Neutrophil (CD11c^low^, CD11b^+^, Ly6C^+^ and Ly6G^+^) **(B)**; monocyte (CD11c^low^, CD11b^+^, Ly6C^+^ and Ly6G^−^) **(C)**; dendritic cell (CD11c^high^, low autofluorescent and MHCII^+^) **(D)**; CD4^+^ T-cell (CD3^+^, CD8^−^, CD4^+^) **(E)** and CD8^+^ T-cell (CD3^+^, CD4^−^, CD8^+^) **(F)** numbers in BALF were determined by flow cytometry. (**G–I**) CXCL1 **(G)**, CCL2 **(H)** and CCL20 **(I)** levels were determined in BALF using ELISA. Results are expressed as mean ± SEM. n = 7–9 mice per group, except the TNFR2 KO mice receiving saline: n = 3 mice. *p < 0.05. BALF inflammatory cell data are representative of two independent experiments (except for BALF neutrophils).

To investigate the specific contribution of each TNF-α receptor to the DEP-induced pulmonary inflammation, we exposed TNFR1 KO, TNFR2 KO and WT mice to saline or 100 µg DEP. DEP-exposed TNFR1 KO mice had similar total BALF cells, neutrophil, monocyte, dendritic cell, CD4^+^ T-cell and CD8^+^ T-cell counts in the BALF compared to WT mice exposed to DEP (Fig. 5A–F). However, in TNFR2 KO mice, DEP-induced BALF monocytes, CD4^+^ T-cells and CD8^+^ T-cells were reduced when compared with WT controls (Fig. 5C,E and F), whereas the number of total BALF cells, neutrophils and dendritic cells was similar between DEP-exposed TNFR2 KO and WT mice (Fig. 5A,B,D).

When compared with DEP-exposed WT controls, the levels of CXCL1, CCL2 and CCL20 in the BALF were decreased in both TNFR1 and TNFR2 KO mice that received DEP (Fig. 5G–I). This decrease was more pronounced in DEP-exposed TNRF2 KO mice.

## Discussion

The cellular and molecular events by which ambient particulate matter such as DEP cause adverse pulmonary health effects remain incompletely known. Using an established murine model of DEP-induced lung inflammation^7–9^, we showed a role for TNF-α signaling in pulmonary inflammation upon DEP exposure. In addition, we identified TNFR2 as the most crucial receptor in DEP-induced pulmonary inflammation.

We demonstrated that exposure to DEP is associated with dose-dependent inflammatory responses in the BALF, including increased numbers of neutrophils, monocytes, dendritic cells, CD4^+^ and CD8^+^ T-cells. These findings in mice mimic the situation in humans, where short-term controlled exposure to DEP was demonstrated to induce accumulation of inflammatory cells in BALF, bronchial biopsies and induced-sputum^3–6^. In addition to the increased cellular inflammation, exposure to DEP is associated with pro-inflammatory cytokine expression in men and in mice^2^. TNF-α is a cytokine with pleiotropic functions that contributes to the pathogenesis of numerous inflammatory diseases^10^. We found increased protein levels of TNF-α and its soluble receptors in BALF of DEP-exposed mice. In line with this, it was previously reported that TNF-α mRNA and protein levels in murine BALF and lung were elevated on exposure to DEP^16–19, 22^.

TNF-α exists in a membrane-bound and soluble form, both of which are biological active^10^. It should be stated that by performing an ELISA on BALF, we only detected the soluble TNF-α form. Membrane shedding of TNF-α and its receptors is mediated by activation of proteases, of which TACE is the most important one^10^. At least in bronchial epithelial cells, it was shown that DEP exposure activated TACE, which resulted in the release of a neutrophil chemoattractant from the epithelium^23^. It is unclear to what extent TNF-α signaling contributed to this, since besides TNF-α, TACE can mediate the shedding of numerous proteins, including epidermal growth factor receptor ligands, which all can contribute to DEP-induced inflammatory responses^23^.

To assess the functional role of TNF-α signaling in pulmonary inflammatory responses on exposure to DEP, we tested TNF-α KO, TNFR-DKO and corresponding WT mice in our model of DEP-induced pulmonary inflammation. We found reduced monocyte, CD4^+^ T-cell and CD8^+^ T-cell numbers, as well as reduced cytokine expression in the BALF of TNF-α KO and TNFR-DKO mice that were exposed to DEP, indicating that TNF-α signaling contributes to the inflammation that is caused by DEP exposure. These findings are in line with observations in models using other pollutants. Indeed, using a model of cigarette-smoke induced lung inflammation, we and others showed that smoke-exposed TNFR-KO mice had an impaired lung inflammation when compared with their air-exposed controls^24, 25^. TNFR-DKO mice were also protected in a model of ozone-induced lung inflammation^26^. Furthermore, treatment with anti-TNF-α antibodies successfully reduced the airway inflammation in smoke- or ozone-exposed animals^27, 28^.

TNF-α exerts its biological functions via interaction with its receptors: TNFR1 and TNFR2. We demonstrate that TNFR2 is the most important receptor in eliciting the DEP-induced lung inflammation, as revealed by decreased inflammatory cell numbers and cytokine expression in DEP-exposed TNFR2 KO mice when compared with DEP-treated WT controls. In contrast, the DEP-induced accumulation of inflammatory cells was similar in TNFR1 KO and WT mice. Levels of DEP-induced CXCL1, CCL2 and CCL20 were however also slightly reduced in absence of TNFR1, thus a minor role for the TNFR1 axis cannot be ruled out. Similar to our current findings, TNFR2 was identified as the most active receptor in cigarette smoke-induced pulmonary inflammation and emphysema^24^. On the other hand, inflammation caused by exposure to silica was reported to be TNFR1-dependent, whereas both the TNFR1 and TNFR2 contributed to subacute ozone-induced lung inflammation^26, 29^. Together, these data suggest that depending on the pollutant, TNF-α can activate different signaling pathways to induce an inflammatory response in the lung. Furthermore, it should be mentioned that other ligands, such as TNF beta (TNF-β, also known as lymphotoxin alpha), can bind the TNF receptors^10^. Since exposure to particulate matter can increase the mRNA expression of TNF-β in lung tissue^30^, it would be worthwhile to investigate how TNF-β signaling relates to pollutant-mediated TNF receptor signaling.

Inhaled DEP targets multiple cell types including hematopoietic cells as well as structural cells such as the epithelium^2^. In line with literature^10^, we demonstrated that TNF-α and TNFR2 were dominantly expressed by lung hematopoietic cells. Given that TNFR2 is preferentially activated by membrane-bound TNF-α^10^, one can speculate that TNF/TNFR2 signalling via hematopoietic cell-cell contacts contributes to the DEP-induced cellular inflammation and cytokines. In contrast, TNFR1 is activated by both membrane-bound and sTNF-α, and is reported to be expressed more uniformly^10^, although we showed the highest expression on CD45^−^ cells. The mild reduction of chemoattractants in DEP-exposed TNFR1 KO mice could therefore suggest a role for TNFR1 in epithelial produced CXCL1, CCL2 and CCL20.

TNF-α binding on its receptors activates a complex network of signaling pathways including NF-κB and mitogen activated protein kinases (MAPK)^10^. Although exposure to DEP is associated with increased activation of these pathways^2^, the specific contribution of DEP-induced TNF-α herein is unclear. Moreover, in an attempt to explain the more crucial role for TNFR2 in our model, it would be interesting to compare the activation of TNFR downstream signaling pathways between WT, TNFR1 and TNFR2 KO mice that were exposed to DEP.

Concerning the DEP-induced neutrophils, Saber *et al*. previously showed no difference in DEP-induced neutrophils in the BALF of TNF-α KO and WT controls^20^, suggesting that TNF-α signaling is not implicated in the DEP-induced neutrophilic inflammation. Although we obtained inconsistent results for DEP-induced neutrophils in preliminary experiments^31^, our current data support the statement of Saber *et al*. that TNF-a signaling is not crucial in the DEP-induced neutrophilic response. Indeed, neutrophils are recruited very early after exposure to DEP or particulate matter (our unpublished findings wherein neutrophils numbers were already increased 6 hours after DEP and^32^), whereas TNF-α levels increased relatively late (i.e. 24 hours) after exposure to DEP^17^. Also upon exposure to ozone, TNF-α signaling only contributed to neutrophilic inflammation induced upon subacute, but not acute ozone exposure^26^. A possible candidate that is important in the (early) DEP-induced neutrophil inflammation could be IL-1β. In this regard, we previously showed that blocking IL-1β or the IL-1R during a single dose of DEP was associated with a strong attenuation of the DEP-induced neutrophilic inflammation^9^.

In controlled chamber studies, exposure to diesel exhaust was shown to increase airway resistance and hyperresponsiveness in healthy persons or individuals with asthma^3, 33–35^. A limitation of our work is that we could not investigate the contribution of TNF-α signaling to DEP-induced AHR, due to the absence of AHR in our model in WT mice. In experimental DEP exposure models, some authors have reported increased AHR^21, 36^, whereas others failed to find AHR in response to DEP^37, 38^. This discrepancy between studies could be due to the (duration of) experimental protocol, the mouse strain that was used, and the method of assessing pulmonary function (i.e. invasive lung function measurements versus noninvasive whole-body plethysmography). On exposure to ozone, airway hyperreactivity to methacholine and acetylcholine was diminished in TNFR1 and TNFR2 KO mice when compared with WT controls^26, 39^.

Besides causing inflammatory responses in the lung, exposure to DEP and ambient particulate matter also associates with adverse cardiovascular events. Indeed, pro-inflammatory cytokines that are produced on exposure to particulate matter can elicit systemic inflammation that increase the risk for cardiovascular diseases^1, 40^. In this regard, human and animal studies have reported increased TNF-α levels in plasma and cardiac tissue in response to diesel exhaust and particulate matter inhalation^15, 41, 42^. Furthermore, blocking TNF-α with a monoclonal antibody attenuated the cardiac dysfunction that was induced by exposure to particulate matter^41^. This suggests that TNF-α signaling can contribute to various adverse health effects that are attributed to particulate matter inhalation.

The clinical use of TNF-α inhibitors in the treatment of obstructive lung diseases such as asthma and chronic obstructive pulmonary disease is controversial and has been reported to be associated with potential adverse side effects. Whereas TNF-α antagonism in a general population of moderate-to-severe asthmatics failed to improve asthma outcome^43^, clinical improvement is suggested in subpopulations of patients with corticosteroid-refractory asthma and high TNF-α expression^44, 45^. At least in mice, TNF-α neutralization restored the therapeutic effects of corticosteroids in glucocorticoid-resisted asthma^46^. Of interest, corticosteroid-refractory asthma has been associated with exposure to particulate air pollutants such as DEP^47^.

In conclusion, we demonstrated a role for TNF-α signaling in DEP-induced lung inflammation. In addition, we showed that TNFR2 is the most important receptor in pulmonary inflammation upon DEP exposure, although a contribution for TNFR1 cannot completely be ruled out. Our data provide insights into the mechanisms by which exposure DEP induces inflammatory responses in the lung. This could be of interest in the search for new tools to protect and treat at-risk individuals from the adverse effects of particulate air pollutants.

## Methods

### Mice

Female C57BL/6 J WT mice were obtained from The Jackson Laboratory. TNF-α, TNFR1 and TNFR2 KO breeding pairs were purchased from The Jackson Laboratory and bred in the animal breeding facility of Ghent University Hospital. TNFR-DKO breeding pairs were obtained from the French National Center for Scientific Research and bred in the animal facility of the Faculty of Medicine and Health Sciences of Ghent University. Animals aged 6–8 weeks were included in the experiments. All *in vivo* manipulations were approved by the Animal Ethical Committee of the Faculty of Medicine and Health Sciences of Ghent University and were performed in accordance with institutional guidelines for animal care.

### DEP instillation

As described previously^7–9^, DEP (SRM 2975) were obtained from the U.S. National Institute for Standards and Technology. DEP were suspended in sterile saline containing 0.05% Tween 80 to a concentration of 2 mg/ml, 1 mg/ml, 0.5 mg/ml or 0.25 mg/ml (i.e. 100 µg (5 mg/kg), 50 µg (2.5 mg/kg), 25 µg (1.25 mg/kg) and 12.5 µg (0.625 mg/kg) dose respectively) and instilled on day 1, 4 and 7. The control group received sterile saline containing 0.05% Tween 80. Prior to instillation, mice were anesthetized with an intraperitoneal ketamine/xylazine injection (70 mg/kg ketamine (Ketamine 1000 CEVA); 7 mg/kg xylazine (Rompun 2%)). Anesthetized mice were held vertically and 50 µl saline or DEP solution was pipetted just above their vocal cords. On day 9, the animals were sacrificed by a lethal dose of intraperitoneal pentobarbital.

### Bronchoalveolar lavage

A tracheal cannula was inserted and BALF was recovered by instillation of 3 × 300 μl HBSS without Ca^2+^ or Mg^2+^ supplemented with 1% BSA (for cytokine and chemokine measurements) and 6 × 500 μl HBSS without Ca^2+^ or Mg^2+^ supplemented with 0.6 mM sodium EDTA. The lavage fractions were pooled. Total cell counts were performed using a Bürcker chamber. Differential cell counts were performed on cytospin preparations after May-Grünwald-Giemsa staining. The remaining cells were used for flow cytometry.

### Flow cytometry

All staining procedures were performed in PBS without Ca^2+^ or Mg^2+^ containing 5 mM EDTA and 1% BSA. To minimize nonspecific bindings, cells were pre-incubated with anti-CD16/CD32 (2.4G2). Cells were labeled with fluorochrome-conjugated monoclonal antibodies against the following cell markers: CD11c (HL3), MHCII (2G9), CD11b (M1/70), Ly6C (AL-21), Ly6G (1A8), CD3 (145-2C11), CD4 (GK1.5) and CD8 (53-6.7). 7-Aminoactinomycin D was used for dead cell exclusion. Data acquisition was performed on a FACSCalibur flow cytometer running CellQuest software. FlowJo software was used for data analysis. Following gating strategy was used: neutrophils were CD11c^−^, CD11b^+^, Ly6C^+^, Ly6G^+^ cells; monocytes were CD11c^-^, CD11b^+^, Ly6C^+^, Ly6G^−^ cells; dendritic cells were CD11c^+^, low autofluorescent, MHCII^+^ cells; CD4^+^ T-cells were CD3^+^, CD4^+^, CD8^−^ cells; CD8^+^ T-cells were CD3^+^, CD8^+^, CD4^−^ cells.

### Cytokine and chemokine measurements

TNF-α, sTNFR1, sTNFR2, CXCL1, CCL2, and CCL20 protein levels in BALF were measured using commercially available ELISA kits (R&D Systems) following the manufacturer’s instructions.

### qRT-PCR

To remove the intravascular pool of cells, lung circulation was rinsed with saline, supplemented with EDTA. Lung tissue were minced and incubated in digestion medium (RPMI 1640 supplemented with 5% FCS, 2 mM L-glutamine, 0.05 mM 2-mercaptomethanol, 100 U/ml penicillin with 100 μg/ml streptomycin, 1 mg/ml collagenase type 2 and 0.02 mg/ml DNase I (grade II from bovine pancreas) for 45 min at 37 °C and 5% CO_2_. RBCs were lysed using ammonium chloride buffer. Hematopoietic (CD45^+^) cells and nonhematopoietic (CD45^−^) lung cells were sorted using an OctoMACS separator and CD45 MicroBeads (according to the manufacturer’s instructions, Miltenyi Biotec). The sorted populations showed >95% purity (data not shown). Next, RNA extraction was performed (miRNeasy Mini Kit), and cDNA (Transcriptor First Strand cDNA synthesis kit) was obtained. Expression of TNF-α, TNFR1 and TNFR2 mRNA, relative to hypoxanthine guanine phosphoribosyl transferase (HPRT) was analyzed using TaqMan gene expression assays. qRT-PCR was performed using a LightCycler 96 system. Data was processed using the standard curve method.

### Airway resistance

Airway resistance was measured in anaesthetized tracheostomized mice using the Flexivent system. Neuromuscular blockade was induced using intravenously injected pancuronium bromide (1 mg/kg). Mice were challenged with increasing doses of carbachol (0, 20, 40, 80, 160, 320, and 640 μg/kg). The resistance (R) of the whole respiratory system (airways, lung and chest wall) was measured.

### Statistical analysis

Statistical analysis was performed with SPSS, version 22.0. Groups were compared using nonparametric tests (Kruskal-Wallis and Mann-Whitney *U*), according to standard statistical criteria. Reported values were expressed as mean ± SEM. P-values under 0.05 were considered as significant.
